# Cloacal Malformation in Female Children: Outcome of Initial Management

**DOI:** 10.12669/pjms.36.2.1095

**Published:** 2020

**Authors:** Naima Zamir

**Affiliations:** Dr. Naima Zamir, Associate Professor, National Institute of Child Health, Karachi, Pakistan

**Keywords:** Cloacal malformation, Colostomy, Female child, Hydrocolpos, Tube vaginostomy

## Abstract

**Objective:**

To document our experience of initial management of Cloacal malformation in female patients.

**Methods:**

A descriptive retrospective study was conducted in the Department of Pediatric Surgery of the National Institute of Child Health Karachi from January 2010 to September 2018. Female patients with diagnosis of Cloacal malformation were included in this study. Data regarding the age at presentation, mode of presentation, clinical features, presence of hydrocolpos, and associated anomalies were noted. Surgical procedures performed in these patients and the outcomes were also documented. Data was analyzed on SPSS Version 20.

**Results:**

Sixty females were included in the study. Age ranged from birth to three years with a median of four days. Patients admitted through emergency were 44 (73.33%) while 16 (26.66%) were admitted through outpatient clinic. Hydrocolpos was found in 15 (25.00%) patients. Five (8.33%) patients had massive abdominal distention and were presented with severe respiratory distress. Among them three had massive hydrocolpos, one patient had pneumoperitoneum secondary to Meckel’s perforation and one patient was having massive colonic dilatation. Hydronephrosis and hydroureter were found in 14 (23.33%) patients, while dilated bladder was found in three (5.00%) patients. After optimization of patients, bowel diversion was done as transverse colostomy in 39 (65.00%) patients, high sigmoid colostomy in 17(28.33%) patients while ileostomy was done in three (5.00%) patients. All patients with hydrocolpos had tube vaginostomy. None of the patients required bladder diversion and urinary tract dilatations were gradually subsided in nine patients in the post-operative period. Associated anomalies were found in 22(36.66%) of cases predominantly of sacral origin. Operative complications were found in 18(30.00%) patients, including stoma site in infection in 12(20.00%) patients, vaginal tube dislodgement in two patients, wrong placement of tube in one patient and vaginostomy stenosis in one case. While bowel stoma retraction occurred in four patients, prolapsed was found in three patients. Renal failure occurred in one patient. In total, 37(61.00%) patients had smooth recovery, 18(30.00%) patients had some complications, while Five (8.33%) patients were expired due to sepsis.

**Conclusions:**

Majority of cloacal malformations present in neonatal life. Initial management is an important step in dealing with these patients. Care must be taken during the abdominal exploration regarding drainage of hydrocolpos and appropriate placement of bowel stoma.

## INTRODUCTION

Cloacal malformation is rare but one of the most complex and challenging varieties of anorectal malformation encountered in the pediatric population. The reported incidence is around 1 in 50,000 life birth and account up to 10% of anorectal malformation.^1^,^2^ By definition, it is a common caudal channel into which urethra, vagina, and rectum terminate and open at the anterior part of the perineum as a single opening.^3^ Embryologically, all three channels fail to develop separately.^4^,^5^ Almost all the cases are of female gender, whereas male cases are very rarely documented.^6^ According to Alberto Pena, many of the cases diagnosed as the rectovaginal fistulas, are actually cloaca, which are misdiagnosed and mismanaged^.7^ The complexity increases when anomaly is associated with hydrocolpos, so is its management. A number of other congenital anomalies are also associated with Cloacal malformation that also affect the clinical outcome.^2^ Vigilant clinical examination of the perineum is very important to identify the correct anomaly and Initial management of the defect is of prime importance in the prognostic outcome and future plan of management.^8^ Here we are sharing our experience of the clinical presentation, initial management and its outcome in patients with Cloacal malformation admitted in our institute.

## METHODS

A retrospective descriptive case series was conducted in one of the surgical units of National institute of child health Karachi from January 2010 to September 2018. Female patients with imperforate anus having single perineal opening in the vestibule with the clinical diagnosis of cloacal malformation were included in the study and underwent initial surgical procedure according to their presentation. Patients with rare complex anomaly of cloacal exstrophy were excluded from the study. Approval from Institutional Ethical Review Board was taken before conducting the study. Detailed counseling of the parents regarding the pathology, management plan and possible outcome was done.

Data regarding the age of the patient at presentation, mode of presentation, associated anomalies, clinical features including presence of abdominal distention, hydrocolpos, respiratory compromise, sepsis, was noted. Ultrasound abdomen and Echocardiography were done before surgery in all patients except in those who had sepsis or respiratory compromise at presentation.

After initial optimization, patients were examined under general anesthesia to confirm the diagnosis and explored for gut diversion. Operative findings, including the distal gut, and internal urogenital tract were noted. Diversion of the systems was made according to the operative findings. Associated anomalies were also dealt if emergency treatment needed. Patients were then followed till four to six weeks for any complications related to wound, bowel stoma, or vaginostomy in case of hydrocolpos that were then managed accordingly. Further evaluation of associated anomalies, Genitography and distal loopography were then advised, followed by genitoscopy under general anesthesia to plan the future definitive treatment. Data regarding the initial management, procedures and their outcome in term of complications were documented. All the data were analyzed on SPSS version 20.

## RESULTS

A total of 60 patients with the diagnosis of Cloacal malformation were included in the study. Age at presentation ranged from day of birth to the 1055 days (three years), with a median age of four days. Patients presented through emergency were 44 (73.33%). All of these patients were neonates, out of which 31 patients had single perineal opening as the only presenting symptom, palpable mass was found in nine patients, massive abdominal distention leading to respiratory compromise was found in five cases including one case of pneumoperitoneum. One patient had esophageal atresia diagnosed on routine clinical examination for neonates. In total, five patients had sepsis at presentation. Patients presented in outpatient clinic were 16 (26.66%), difficulty in passing stool was the presenting symptom in 11 patients, and three patients were presented with palpable abdominal mass while two patients were presented with ambiguity in external genitalia. With high index of suspension perineal examination was done in these patients, which revealed a single perineal opening. In total, the ultrasound abdomen-pelvis and echocardiography were done preoperatively on 55 (91.66%) patients. Ultrasound showed hydrocolpos in 11 patients while in 14 (23.33%) patients associated hydronephrosis and hydro ureter were documented.

After optimization, 5 (8.33%) patients, with massive abdominal distention and respiratory compromise were explored in emergency without prior Ultrasound and echocardiography. Among them, three patients had massive hydrocolpos, one patient had massive dilatation of colon and one patient had Meckel’s diverticulum perforation. The other 55 patients underwent elective surgery. Examination of perineum under anesthesia prior to abdominal exploration showed a very short common channel (<1cm) in one patient.

Bowel diversion was done as transverse colostomy in 39 (65.00%) patients and sigmoid colostomy was done in 17(28.33%) patients. Three patients (05.00%) had ileostomies, one for perforated Meckel’s diverticulum and two for pouch colon. In patients with low confluence, anoplasty served the purpose. Regarding hydrocolpos, tube vaginostomy was done in 14 patients. Three (05.00%) patients had dilated floppy bladder. They were drained per urethra and no urinary diversion was needed.

The associated congenital anomalies were found 41 in 22 (36.66%) patients as shown in Table-I. The most common were the sacral anomalies in 12 (20.00%) patients, followed by renal anomalies in 10 (16.66%) patients. While genital tract anomalies were found nine (15.00%) cases including five septate vaginas, three bicornuate uteri, two cases of enlarged Clitoris and, in one case there was absent vagina. Associated esophageal atresia with trachea-esophageal fistula and malrotation were managed in the same settings of stoma formation in emergency. However, none of the patients were with associated major cardiac anomaly.

**Table-I:** Associated Anomalies (N=60).

Anomalies	Frequencies	Percentages %
Sacral anomalies	12	20.00
Genital anomalies	9	15.00
Single kidney	8	13.33
Ectopic kidney	2	3.33
Down syndrome	2	3.33
Pouch colon	2	3.33
Esophageal Atresia	1	1.66
Labial swelling	1	1.66
Malrotation	1	1.66
Meckel’s diverticulum	1	1.66
Anotia	1	1.66
Cleft lip	1	1.66

Total	41	

Postoperatively total of 18(30.00 %) patients developed 24 complications within one month of surgery. Stoma site infection was found in 12 patients. All were managed conservatively. Bowel stoma retraction was found in four patients, who required redo stoma while stoma prolapse occurred in three patients with one of these patients requiring the revision of stoma. As far as Vaginostomy is concerned, five patients needed revision. As in two cases, the tube was dislodged in peritoneal cavity, and in one case it was mistakenly placed in ileum. In one case vaginostomy got stenosis leading to incomplete drainage of fluid. In one case, the hydrocolpos was missed in initial exploration resulted in renal failure due to constant pressure on the urinary tract. It was revealed later on an ultrasound as cystic area behind the bladder. In that case, after optimization vaginostomy was made. Hydronephrosis and hydroureter gradually subsided postoperatively in nine patients.

**Table-II:** Post-Operative Outcome (N=60).

Complications	Frequencies	Percentages
Wound infection	12	20.00%
Bowel stoma retracted	4	6.66
Bowel stoma prolapse	3	5.00
Vaginostomy tube dislodged	3	5.00
Vaginostomy stenosis	1	1.66
Renal Failure	1	1.66
Expire	5	8.33

Five patients (8.33%) expired, due to possible uncontrolled sepsis that included two patients with massive hydrocolpos and one patient who also had esophageal atresia operated in the same setting. A total of 37 (61.66%) patients had a smooth course of recovery while 18 (30.00%) patients had some complication that required to be taken care of before future evaluation and plan of management.

## DISCUSSION

Cloacal malformation has a wide spectrum of defects, having greater management challenges.^4^,^9^ Presence of abdominal mass due to hydrocolpos and urological pathologies, and the other associated anomalies make its diagnosis and management even more complex.^10^ The initial management of the Cloacal malformation plays an important role in guiding the future definitive plan and outcome. The goal of this management after stabilization of the patient and identification of associated anomalies is the drainage of genitourinary systems and diversion of the bowel.^11^ Antenatal diagnosis of Cloacal malformation with ultrasound has been documented in literature, especially if associated with hydrocolpos.^12^,^13^ None of the patients in the current study had an antenatal diagnosis of this anomaly. This may be related to lack of expertise or advanced facilities to detect the anomaly in antenatal ultrasound. Postnatal diagnosis is possible clinically by the presence of a single opening in the vestibule of vagina with abdominal distention, urinary retention, and inability to pass meconium being the commonest presenting features.^2^

Majority of patients in the current series were presented in emergency at neonatal age with typical picture while five patients among them also had complex symptoms like severe respiratory distress and sepsis. Late presentation has also been documented in literature, may be due to lack of awareness, delayed symptoms, and inaccessibility to medical facilities.^14^ We received five patients beyond neonatal age that is between five months to three years. They presented in outpatient clinic with constipation, a silent lower abdominal mass or even ambiguity of external genital appearance and had been extensively investigated similar to documented in literature.^2^ This highlights the importance of a high index of suspicion and clinical examination of the perineum that can obviate the extended investigations.

Although preoperative evaluation for associated anomalies is the recommended step in the planning of management, it may be deferred in cases where emergency surgery is required.^15^ In our series, five patients were explored without prior investigations for associated anomalies. The reported frequency of hydrocolpos in Cloacal malformation is around 28.5% which is found to be 25% in the current series.^16^ Hydrocolpos can cause pressure effects on the urinary system leading to obstruction, dilatation and damage of renal system that may cause infection, perforation or even sepsis.^17^ In the current study, patients had unilateral or bilateral hydronephrosis or dilated urinary bladder, the conditions subsided after bowel and genital tract decompression suggestive of caused by the pressure effect in majority of cases and thus no urinary diversion was needed in our patients. This was similar to what is documented in literature.^9^ The diagnosis of hydrocolpos was missed in one of our patients on initial exploration. Thus there was a delay in its management that resulted in renal failure in that particular case.

Emergency management of other anomalies along with the exploration for bowel and genital diversion increases the morbidity and mortality, especially if associated sepsis at presentation. Like in our series of patients, one patient with esophageal atresia expired secondary to overwhelming sepsis in post-operative period. Contrary to documented literature, no major cardiac anomaly was found in the current study.^8^,^18^ On initial abdominal exploration, a thorough examination of whole gut and internal urogenital organs is of prime importance to formulate the immediate diversion plan and future management strategy.^19^ Severe variety of Pouch colon, short gut anomalies mandate the formation of ileostomy instead of colostomy like we did in two of our patients.^20^ Placement of the bowel stoma is one of the important and crucial steps in the management of cloacal malformations leaving adequate distal gut length to be used in future for definitive pull-through procedures or vaginal reconstruction and also at the same time not interfering during the definitive surgical procedures.^2^,^14^ Keeping this fact in mind right transverse colostomy was opted in majority of our patients while sigmoid colostomy was done in patients with low anal confluence. The documented drainage procedures for hydrocolpos are abdominal cutaneous Vaginostomy, tube vaginostomy or intermittent perineal catheterization.^16^ As per institutional protocol; tube vaginostomy was done in all the patients with hydrocolpos for the drainage and prevention of re-accumulation of fluid, thus avoiding the complications like infection or perforation besides the pressure effect on urinary tract. Bicornuate uterus and double vagina with hydrocolpos are common reported genital anomalies associated cloaca and these could be used for future reconstruction of genital tract.^16^,^18^,^21^ Similar findings were also observed in our study.

Regarding operative complications found in our patients, these were mistakenly placed vaginostomy tube in the ileum instead of vagina and the dislodgement of the tube in the peritoneal cavity while draining the hydrocolpos. Thus, we ended in re-exploration of these patients. This probably occurs due to difficult anatomical demarcation between dilated vagina, bladder and bowel.^16^ Therefore, careful identification and confirmation of the vagina is very important for the placement of vaginostomy tube. In post-operative period stoma site infection was found to be the most common complication in the current series. This could be related to emergency exploration of neonates in compromised state superimposed by their yet underdeveloped immune system. The other complications found in the current series were vaginostomy stenosis and bowel stoma retraction or prolapse.^22^,^23^ Severe sepsis at the time of presentation was the major cause of mortality in our series.^24^ The mortality was further increased if superimposed by the associated anomalies.

### Limitations of the study

It is the unavailability of neonatal sized endoscope, especially emergency situation which delayed the evaluation of common channel of cloaca and the definitive procedure.

Majority of cloacal malformations present in neonatal life even with superimposed sepsis. Initial evaluation and surgical management is an important step in dealing with these patients. Care must be taken during the abdominal exploration regarding drainage of hydrocolpos and appropriate placement of bowel stoma to avoid complications and to apposite future management.

## CONCLUSION

Majority of cloacal malformations present in neonatal life even with superimposed sepsis. Initial evaluation and surgical management is an important step in dealing with these patients. Care must be taken during the abdominal exploration regarding drainage of hydrocolpos and appropriate placement of bowel stoma to avoid complications and to apposite future management.
